# ﻿Morpho-molecular analyses reveal two novel species and two new records of Dictyosporiaceae (Pleosporales) from Dulongjiang River in northwestern Yunnan Province, China

**DOI:** 10.3897/mycokeys.117.145587

**Published:** 2025-04-30

**Authors:** Ying Wang, D. Jayarama Bhat, Dan-Feng Bao, Hong-Wei Shen, Yuan Feng, Zong-Long Luo

**Affiliations:** 1 College of Agriculture and Biological Science, Dali University, Dali 671003, Yunnan, China Dali University Dali China; 2 Department of Botany and Microbiology, College of Science, King Saud University, P.O. Box 2455, Riyadh 11451, Saudi Arabia King Saud University Riyadh Saudi Arabia; 3 Vishnugupta Vishwavidyapeetam, Ashoke, Gokarna 581326, India Vishnugupta Vishwavidyapeetam Ashoke India; 4 Engineering and Research Center for Southwest Biopharmaceutical Resource of National Education Ministry of China, Guizhou University, Guiyang, 550025, Guizhou, China Guizhou University Guiyang China

**Keywords:** Dothideomycetes, lignicolous freshwater fungi, new species, phylogeny, taxonomy

## Abstract

Yunnan Province is rich in freshwater resources, providing a favorable environment for freshwater fungi. As a result, numerous new species have been described in recent years, further supporting the extensive diversity of freshwater fungi in this region. During an investigation of lignicolous freshwater fungi from the Dulongjiang River, six fresh isolates of Dictyosporiaceae were obtained. The new collection includes two new species (*Dictyocheirosporasplendida* and *Jalapriyaguttulata*), two new records (*D.nabanheensis* and *Dictyosporiumhughesii*), and one known species (*Dictyosporiumduliujiangense*). They are introduced based on morphological evidence and molecular phylogenetic analysis of combined ITS, LSU, and *tef*1-α sequence data. Detailed descriptions and illustrations of these species are provided, and morphological comparison with similar taxa is discussed.

## ﻿Introduction

Lignicolous freshwater fungi are key decomposers in freshwater ecosystems (Rebeca et al. 2021; Tamam et al. 2021). Recent studies have shown that freshwater fungi globally encompass 13 phyla in the Kingdom Fungi (Calabon et al. 2022). Ascomycota as the dominant group, and Sorsariomycetes leading, followed by Dothideomycetes, is a trend observed in China, especially in Yunnan (Calabon et al. 2022; Shen et al. 2022a). Dothideomycetes represents a major taxonomic group, characterized by bitunicate asci, typically with fissitunicate dehiscence, and many species have been collected from freshwater habitats (Dong et al. 2020). Recent data indicated that freshwater dothideomycetous species reside in six orders, 43 families, and 145 genera, with 46 genera exclusively documented from freshwater environments (Dong et al. 2020; Calabon et al. 2022, 2023).

Boonmee et al. (2016) established Dictyosporiaceae to accommodate most cheirosporous hyphomycetous genera, with *Dictyosporium* as the type genus. Tennakoon et al. (2023) documented that the Dictyosporiaceae comprises 154 species. However, *Dictyocheirosporahimachalensis* was not included in the revised version (Sushma et al. 2022). Presently, the species number in Dictyosporiaceae has increased to 178 (Phookamsak et al. 2019; Dong et al. 2023; Liu et al. 2023; Zhang et al. 2023; Feng et al. 2024; Han et al. 2024; Liu et al. 2024; Phookamsak et al. 2024; Shu et al. 2024; Sun et al. 2024; Wang et al. 2024; Wu et al. 2024; Zhang et al. 2024a; Zhang et al. 2024b; Du et al. 2025). These taxa subsist as saprobes on decaying wood and plant debris in aquatic and terrestrial habitats worldwide (Boonmee et al. 2016; Fu et al. 2021; Shen et al. 2022b). Currently, Dictyosporiaceae comprises 23 genera, with most of the asexual morphic members of this family being hyphomycetous and characterized by cheiroid, digitate, palmate, and/or dictyosporous, and pale brown to dark brown conidia. These include the following: *Aquadictyospora*, *Aquaticheirospora*, *Cheirosporium*, *Dendryphiella*, *Dictyocheirospora*, *Dictyopalmispora*, *Dictyosporium*, *Digitodesmium*, *Jalapriya*, *Kamatia*, *Neodendryphiella*, *Neodigitodesmium*, *Neogregarithecium*, *Paradictyocheirospora*, *Pseudodictyosporium*, and *Vikalpa*, and six coelomycetous genera, *viz.*, *Immotthia*, *Pseudocoleophoma*, *Pseudoconiothyrium*, *Pseudocyclothyriella*, *Sajamaea*, and *Verrucoccum*. Whereas five genera are known to have sexual morphs, viz., *Dictyosporium*, *Gregarithecium*, *Immotthia*, *Pseudocoleophoma*, and *Verrucoccum*. Notably, *Dictyosporium* exhibits both sexual and asexual hyphomycetous morphs, while *Immotthia*, *Pseudocoleophoma*, and *Verrucoccum* display sexual and asexual coelomycetous morphs (https://www.outlineoffungi.org/; Boonmee et al. 2016; Jiang et al. 2021; Shen et al. 2022b; Wijayawardene et al. 2022; Tennakoon et al. 2023; Shu et al. 2024).

*Dictyocheirospora* was established by Boonmee et al. (2016) with *D.rotunda* as the type species. This genus is characterized by dark sporodochial colonies that produce aeroaquatic cheiroid, doliiform to cylindrical conidiogenous cells, with or without hooked apices, with or without appendages, and non-complanate or cylindrical conidia (Boonmee et al. 2016). The genus comprises 32 species (Hyde et al. 2017; Jayasiri et al. 2019; Shen et al. 2022b; Sushma et al. 2022; Liu et al. 2023; Shu et al. 2024; Sun et al. 2024; Wang et al. 2024; Wu et al. 2024), with eight being transferred from *Dictyosporium* (Boonmee et al. 2016; Yang et al. 2018).

Corda (1836) established *Dictyosporium* with *Di.elegans* as the type species. This genus is characterized by dark brown, subglobose ascomata, bitunicate cylindrical asci, and hyaline, fusiform uniseptate ascospores with or without a sheath, and sporodochial or effuse conidiomata, micronematous and mononematous conidiophores (or absent), and cheiroid, digitate complanate conidia with several parallel rows of cells (Prasher et al. 2015; Boonmee et al. 2016; Silva et al. 2016; Yang et al. 2018; Shen et al. 2022b). Dubey (2022) documented 13 species of *Dictyosporium*, which have already been reassigned to other genera (Boonmee et al. 2016; Yang et al. 2018). The genus comprises 58 species that are mainly found on dead wood and decaying leaves in terrestrial and freshwater environments (Dubey 2022; Shen et al. 2022b; Liu et al. 2023; Shu et al. 2024; Zhang et al. 2024b).

*Jalapriya* was introduced by Boonmee et al. (2016) with *J.pulchra* as the type species. The members of this genus are characterized by dark brown to black colonies, acrogenous, solitary, and cheiroid conidia (Boonmee et al. 2016). Presently, six species are accepted in the genus, viz., *J.apicalivaginatum*, *J.aquaticum*, *J.cheirospora*, *J.inflata*, *J.pulchra*, and *J.toruloides* (Du et al. 2025).

Dulongjiang River is one of the six major river systems in Yunnan Province, China. Originating from Xizang Province, it flows through the northwestern Yunnan Province, China, and then enters into Myanmar. We are carrying out a survey of species diversity of lignicolous freshwater fungi in the Dulongjiang River basin, and this is the first in a series of papers to appear on these fungi. In this study, six fresh isolates of Dictyosporiaceae were obtained from submerged wood in Dulongjiang River and are identified as five species, two of which are new. Descriptions and illustrations are provided for these species. In addition, an updated backbone tree of Dictyosporiaceae is performed to clarify species relationships and support the establishment of novel taxa.

## ﻿Materials and methods

### ﻿Sample collection, specimen examination, and isolation

Submerged decaying woods were collected from the Dulongjiang River in Yunnan Province, China, in May 2023 and taken to the laboratory in Ziploc plastic bags. The sample incubation, examination, and morphological studies were carried out following the methods described by Luo et al. (2018a). Macromorphological characteristics were observed using an Optec SZ 760 compound stereomicroscope (Chongqing Optec Instrument Co., Ltd., Chongqing, China). The morphologies of colonies on original substrates were photographed with a Nikon SMZ1000 stereo zoom microscope (Nikon Corporation, Tokyo, Japan). Semi-permanent slides were observed using a Nikon ECLIPSE Ni-U compound stereomicroscope (Nikon Corporation, Tokyo, Japan) and photographed micro-morphological characteristics.

Single spore isolations were carried out based on the method described by Luo et al. (2018a) and Senanayake et al. (2020). Using a sterilized toothpick, a small amount of conidia was picked and streaked on potato dextrose agar (PDA) or water agar (WA) plates to spread the conidia. The plates were incubated at room temperature for approximately 12 hours to facilitate the formation of germ tubes. Germinated conidia were aseptically transferred to new PDA plates and incubated in an incubator room at 22 °C.

Specimens (dry wood with fungal material) were deposited in the Herbarium of Cryptogams Kunming Institute of Botany, Chinese Academy of Sciences (KUN-HKAS), Kunming, China. Living cultures were deposited in the Kunming Institute of Botany Culture Collection Center, Kunming, China (KUNCC). Fungal Names number (FN) (https://nmdc.cn/fungalnames/) for the new species was registered. New species were established following the recommendations outlined by Chethana et al. (2021).

### ﻿DNA extraction, PCR amplification, and sequencing

Fungal mycelium was scraped from the surface of colonies grown on PDA and transferred to a 1.5 mL centrifuge tube. The Trelief TM Plant Genomic DNA Kit (TSP101-50) was used to extract DNA from the ground mycelium according to the manufacturer’s instructions. The ITS, LSU, and *tef*1-α gene regions were amplified using the primer pairs ITS5/ITS4, LR0R/LR7, and 983F/2218R, respectively (Vilgalys and Hester 1990; Liu et al. 1999). The amplification was performed in a 25 μL reaction volume containing 9.5 μL deionized water, 12.5 μL 2 × Taq PCR Master Mix with blue dye (Sangon Biotech, Shanghai, China), 1 μL of DNA template, and 1 μL of each primer (10 μM). The amplification condition for ITS, LSU, and *tef*1-α followed Bao et al. (2022) and Shen et al. (2021). PCR amplification was confirmed on 1% agarose electrophoresis gels. Purification and sequencing of PCR products were sent for sequencing at Tsingke Biological Engineering Technology and Services Company, Yunnan, China.

### ﻿Molecular phylogenetic analyses

#### ﻿Sequence alignment

Sequences were assembled using BioEdit v. 7.2.5.0 (Dagona 1999). A BLAST search in the National Center of Biotechnology Information (NCBI) was performed on sequences with high similarity indices to find the closest matches with taxa. The ITS, LSU, and *tef*1-α used for phylogenetic analysis are selected based on the preliminary identification results and the related publications (Shen et al. 2022b; Tennakoon et al. 2023; Shu et al. 2024). Closely relevant sequences were downloaded from the National Center of Biotechnology Information (https://www.ncbi.nlm.nih.gov/). All consensus sequences and the reference sequences were automatically aligned with MAFFT v. 7.0 (http://mafft.cbrc.jp/alignment/server/index.html, accessed on 11 December 2024; Katoh et al. 2019). The sequence dataset was combined using SequenceMatrix v.1.7.8 (Vaidya et al. 2011). The alignment formats were converted to Phylip and Nexus formats by AliView (Larsson 2014).

Maximum likelihood (ML) analysis was performed by setting RaxML-HPC2 on XSEDE (8.2.12) (Stamatakis 2006; Stamatakis et al. 2008) in the CIPRES Science Gateway (https://www.phylo.org/portal2/login!input.action). The final RAxML search was conducted using the GTRGAMMA+I model (Nylander 2004; Ronquist et al. 2012). Maximum likelihood bootstrap support was calculated from 1000 bootstrap replicates (https://www.phylo.org/portal2).

Bayesian analysis was performed using MrBayes v. 3.2 (Ronquist et al. 2012). The evolution model was estimated using MrModeltest 2.3 (Nylander 2004), and the GTR+I+G model is the best-fit model of ITS, LSU, and *tef*1-α. Posterior Probabilities (PP) (Rannala and Yang 1996) were performed by Markov Chain Monte Carlo sampling (MCMC) in MrBayes v.3.1.2 (Liu et al. 2012). Six simultaneous Markov chains were run for 100 million generations, and trees were sampled every 10000^th^. The first 25% trees representing the burn-in phase of the analyses were discarded, and the remaining 75% trees (post-burning) were used for calculating posterior probabilities (PP) in the majority rule consensus tree. Phylogenetic trees were printed with FigTree v.1.4.4. (http://tree.bio.ed.ac.uk/software/figtree/), while editing and typesetting were achieved using Adobe Illustrator (AI) (Adobe Systems Inc., California, USA). Newly generated sequences in this study were submitted to GenBank (Table 1).

**Table 1.** GenBank and culture collection accession numbers of species used in the phylogenetic analyses. The newly generated sequences are indicated in red, and the type strains are indicated in bold.

Species	Voucher/Culture	GenBank accession number		
		ITS	LSU	*tef*1-α
***Aquadictyosporaclematidis***	**MFLUCC 17**-**2080**	**MT310592**	**MT214545**	**MT394727**
***Aquadictyosporalignicola***	**MFLUCC 17**-**1318**	**MF948621**	**MF948629**	**MF953164**
***Aquaticheirosporalignicola***	**HKUCC 10304**	**AY864770**	**AY736378**	–
***Cheirosporiumtriseriale***	**HMAS 180703**	**EU413953**	**EU413954**	–
***Dendryphiellaeucalyptorum***	**CBS 137987**	**KJ869139**	**KJ869196**	–
***Dendryphiellafasciculata***	**MFLUCC 17**-**1074**	**MF399213**	**MF399214**	–
***Dendryphiellaparavinosa***	**CBS 141286**	**KX228258**	**KX228309**	–
***Dendryphiellaphitsanulokensis***	**MFLUCC 17**-**2513**	**MG754400**	**MG754401**	–
***Dendryphiellavariabilis***	**CBS 584.96**	**LT963453**	**LT963454**	–
*Dictyocheirosporaacaciae*	SDBR-CMU454	OP965332	OP965372	OQ000838
*Dictyocheirosporaacaciae*	SDBR-CMU455	OP965333	OP965373	OQ000839
***Dictyocheirosporaaquadulcis***	**MFLUCC 17**-**2571**	**MK634545**	**MK634542**	–
*Dictyocheirosporaaquadulcis*	MFLUCC 22-0095	OP526634	OP526644	OP542236
***Dictyocheirosporaaquatica***	**KUMCC 15**-**0305**	**KY320508**	**KY320513**	–
***Dictyocheirosporabannica***	**KH 332**	**LC014543**	**AB807513**	**AB808489**
*Dictyocheirosporabannica*	MFLUCC 16-0874	MH381765	MH381774	–
*Dictyocheirosporacheirospora*	KUMCC 17-0035	MF177035	MF177036	–
***Dictyocheirosporachiangmaiensis***	**MFLUCC 22**-**0097**	**OP526630**	**OP526640**	**OP542232**
***Dictyocheirosporaclematidis***	**MFLUCC 17-2089**	**MT310593**	**MT214546**	**MT394728**
***Dictyocheirosporagarethjonesii***	**MFLUCC 16**-**0909**	**KY320509**	**KY320514**	–
*Dictyocheirosporagarethjonesii*	DLUCC 0848	MF948623	MF948631	MF953166
*Dictyocheirosporagigantica*	BCC 11346	DQ018095	–	–
*Dictyocheirosporaheptaspora*	MFLUCC 22-0096	OP526635	OP526645	OP542237
*Dictyocheirosporaheptaspora*	CBS 396.59	DQ018090	–	–
*Dictyocheirosporaindica*	MFLUCC 15-0056	MH381763	MH381772	MH388817
***Dictyocheirosporalithocarpi***	**MFLUCC 17**-**2537**	**MK347781**	**MK347999**	–
***Dictyocheirosporametroxylonis***	**MFLUCC 15**-**0028a**	**MH742321**	**MH742313**	–
***Dictyocheirosporametroxylonis***	**MFLUCC 15**-**0028b**	**MH742322**	**MH742314**	**MH764301**
*Dictyocheirosporamultiappendiculata*	KUNCC 22-10736	OP526633	OP526643	OP542235
***Dictyocheirosporamultiappendiculata***	**KUNCC 22**-**10734**	**OP526632**	**OP526642**	**OP542234**
***Dictyocheirosporanabanheensis***	**MFLUCC 17**-**0562**	**MH388340**	**MH376712**	**MH388375**
*Dictyocheirosporanabanheensis*	MFLUCC 22-0094	OP526637	OP526647	OP542239
*Dictyocheirosporanabanheensis*	KUNCC 23-15886	PQ309051	PQ309042	PQ346446
***Dictyocheirosporapandanicola***	**MFLUCC 16**-**0365**	**MH388341**	**MH376713**	**MH388376**
***Dictyocheirosporapseudomusae***	**yone 234**	**LC014550**	**AB807520**	**AB808496**
***Dictyocheirosporarotunda***	**MFLUCC 14**-**0293**	**KU179099**	**KU179100**	–
*Dictyocheirosporarotunda*	MFLUCC 17-0222	MH381764	MH381773	MH388818
*Dictyocheirosporarotunda*	GZCC 19-0436	OQ842723	MW133811	OQ850744
*Dictyocheirosporarotunda*	GZCC 19-0429	OQ842724	MW133814	OQ850745
*Dictyocheirosporarotunda*	MFLUCC 17-1313	MF948625	MF948633	MF953168
***Dictyocheirosporasplendida***	**KUNCC 23-15971**	**PQ309052**	**PQ309043**	**PQ346447**
***Dictyocheirosporasuae***	**KUNCC 22**-**12424**	**OP526631**	**OP526641**	**OP542233**
***Dictyocheirosporasubmersa***	**ZHKUCC 24**-**0001**	**PP326193**	**PP326216**	**PP333113**
*Dictyocheirosporasubramanianii*	BCC 3503	DQ018094	–	–
***Dictyocheirosporataiwanense***	**MFLUCC 17**-**2654**	**MK495821**	**MK495820**	–
***Dictyocheirosporathailandica***	**MFLUCC 18**-**0987**	**MT627734**	**MN913743**	–
*Dictyocheirosporavinaya*	MFLUCC 14–0294	KU179102	KU179103	–
***Dictyocheirosporaxishuangbannaensis***	**KUMCC 17**-**0181**	**MH388342**	**MH376714**	**MH388377**
***Dictyosporiumalatum***	**ACC 34953**	**NR077171**	**DQ018101**	–
***Dictyosporiumappendiculatum***	**MFLUCC 17**-**2259**	**MH388343**	**MH376715**	–
***Dictyosporiumaquaticum***	**MF1318**	**KM610236**	–	–
*Dictyosporiumbulbosum*	yone 221	LC014544	AB807511	AB808487
*Dictyosporiumdigitatum*	KH 401	LC014545	AB807515	AB808491
*Dictyosporiumdigitatum*	yone 280	LC014547	AB807512	AB808488
***Dictyosporiumduliujiangense***	**GZCC 19**-**0426**	**OQ842725**	**MW133815**	**OQ850746**
*Dictyosporiumduliujiangense*	KUNCC 23-15949	PQ309055	PQ309046	PQ346450
***Dictyosporiumelegans***	**NBRC 32502**	**DQ018087**	**DQ018100**	–
***Dictyosporiumguangdongense***	**ZHKUCC 24**-**0002**	**PP326190**	**PP326213**	–
***Dictyosporiumguttulatum***	**MFLUCC 16**-**0258**	**MH388345**	**MH376717**	**MH388379**
*Dictyosporiumhongkongensis*	KUMCC 17-0268	MH388346	MH376718	MH388380
*Dictyosporiumhughesii*	K 1847	LC014548	AB807517	AB808493
*Dictyosporiumhughesii*	KUNCC 23-15923	PQ309056	PQ309047	–
***Dictyosporiumkrabiense***	**MFLU 16**-**1890**	–	**MH376719**	**MH388381**
***Dictyosporiummeiosporum***	**MFLUCC 10**-**0131**	**KP710944**	**KP710945**	–
***Dictyosporiummuriformis***	**GZCC 20**-**0006**	**MT002304**	**MN897834**	**MT023011**
***Dictyosporiumnigroapice***	**MFLUCC 17**-**2053**	**MH381768**	**MH381777**	**MH388821**
*Dictyosporiumnigroapice*	BCC 3555	DQ018085	–	–
***Dictyosporiumolivaceosporum***	**KH 375**	**LC014542**	**AB807514**	**AB808490**
*Dictyosporiumpalmae*	CBS-H 22129	–	KX555648	–
***Dictyosporiumpandanicola***	**MFLU 16**-**1886**	**MH388347**	**MH376720**	**MH388382**
***Dictyosporiumsexualis***	**MFLUCC 10**-**0127**	**KU179105**	**KU179106**	–
*Dictyosporium* sp.	MFLUCC 15-0629	MH381766	MH381775	MH388819
***Dictyosporiumstellatum***	**CCFC 241241**	**NR 154608**	**JF951177**	–
***Dictyosporiumstrelitziae***	**CBS 123359**	**NR 156216**	**FJ839653**	–
*Dictyosporiumtetrasporum*	K 2865	LC014551	AB807519	AB808495
***Dictyosporiumthailandicum***	**MFLUCC 13**-**0773**	**KP716706**	**KP716707**	–
***Dictyosporiumtratense***	**MFLUCC 17**-**2052**	**MH381767**	**MH381776**	**MH388820**
***Dictyosporiumtubulatum***	**MFLUCC 15**-**0631**	**MH381769**	**MH381778**	**MH388822**
*Dictyosporiumtubulatum*	MFLUCC 17-2056	MH381770	MH381779	–
***Dictyosporiumvariabilisporum***	**ZHKUCC 24**-**0003**	**PP326192**	**PP326215**	**PP333112**
***Dictyosporiumwuyiense***	**CGMCC 3.18703**	**KY072977**	–	–
***Dictyosporiumzhejiangense***	**MW–2009a**	**FJ456893**	–	–
***Digitodesmiumaquaticum***	**MFLU 22**-**0203**	**OP749872**	**OP749877**	**OP756064**
***Digitodesmiumbambusicola***	**CBS 110279**	**DQ018091**	**DQ018103**	–
***Digitodesmiumchiangmaiense***	**HKAS 102163**	–	**MK571766**	–
***Digitodesmiumchishuiense***	**GZCC 20**-**0510**	**OP377808**	**OP377907**	**OP472990**
*Digitodesmiumpolybrachiatum*	COAD 3174	MW879318	MW879316	MW890262
*Digitodesmiumpolybrachiatum*	COAD 3175	MW879319	MW879317	MW890263
*Digitodesmium* sp.	BRC 10038	MK405235	MK405233	MK405231
*Digitodesmium* sp.	BRC 10037	MK405234	MK405232	MK405230
***Digitodesmiumtectonae***	**NFCCI 4878**	**MW854646**	**MW854647**	**MW854832**
***Gregaritheciumcurvisporum***	**MFLUCC 13**-**0853**	**KX364281**	**KX364282**	–
*Gregaritheciumcurvisporum*	K 922	AB809644	AB807547	–
*Immotthiaatrograna*	Z-Myc-64283	MW489540	–	–
***Immotthiabambusae***	**HKAS 112012AI**	**MW489455**	**MW489450**	**MW504646**
***Immotthiabambusae***	**HKAS 112012C**	**MW489458**	**MW489453**	**MW504648**
***Jalapriyaapicalivaginatum***	**HKAS 115801**	**MZ621167**	**MZ621168**	–
*Jalapriyaaquaticum*	DLUCC 2351	MZ621151	MZ621165	–
***Jalapriyaaquaticum***	**HKAS 115807**	**MZ621152**	**MZ621169**	**MZ851995**
***Jalapriyaguttulata***	**KUNCC 23-15861**	**PQ309053**	**PQ309044**	**PQ346448**
*Jalapriyaguttulata*	KUNCC 23-14576	PQ309054	PQ309045	PQ346449
*Jalapriyainflata*	NOU 3855	JQ267362	JQ267363	–
***Jalapriyapulchra***	**MFLUCC 15**-**0348**	**KU179108**	**KU179109**	–
*Jalapriyapulchra*	MFLUCC 17-1683	MF948628	MF948636	MF953171
*Jalapriyatoruloides*	CBS 209.65	DQ018093	DQ018104	–
***Neodendryphiellamali***	**FMR 16561**	**LT906655**	**LT906657**	–
***Neodendryphiellamichoacanensis***	**FMR 16098**	**LT906660**	**LT906658**	–
***Neodendryphiellatarraconensis***	**FMR 16234**	**LT906659**	**LT906656**	–
***Neodigitodesmiumcheirosporum***	**UESCC 22**-**0020**	**ON595714**	**ON595713**	**ON595700**
***Nigrogranamycophila***	**CBS 141478**	**NR 147654**	–	–
*Nigrogranamycophila*	MF6	KX650554	–	KX650526
*Periconiaigniaria*	CBS 379.86	LC014585	AB807566	AB808542
*Periconiaigniaria*	CBS 845.96	LC014586	AB807567	AB808543
*Pseudocoleophomabauhiniae*	MFLUCC 17-2586	MK347736	MK347953	MK360076
*Pseudocoleophomabauhiniae*	MFLUCC 17-2280	MK347735	MK347952	MK360075
***Pseudocoleophomacalamagrostidis***	**K 3284**	**LC014592**	**LC014609**	**LC014614**
*Pseudocoleophomaflavescen*	CBS 178.93	–	GU238075	–
***Pseudocoleophomapolygonicola***	**K 731**	**AB809634**	**AB807546**	**AB808522**
***Pseudocoleophomapuerensis***	**ZHKUCC 22 0204**	**NR 184995**	**NG154047**	**OP321568**
*Pseudocoleophomapuerensis*	ZHKUCC 22 0205	OP297800	OP297770	OP321569
*Pseudocoleophomarhapidis*	ZHKUCC 21 0124	ON244664	ON244661	–
*Pseudocoleophomarhapidis*	ZHKUCC 22 0004	ON244665	ON244662	–
*Pseudocoleophomarusci*	MFLUCC 16-1444	MT185549	MT183514	–
***Pseudocoleophomatyphicola***	**MFLUCC 16**-**0123**	**KX576655**	**KX576656**	–
*Pseudocoleophomazingiberacearum*	NCYUCC 19-0052	MN615939	MN616753	MN629281
*Pseudocoleophomazingiberacearum*	NCYUCC 19-0053	MN615940	MN616754	MN629282
***Pseudoconiothyriumbroussonetiae***	**CBS 145036**	**NR 163377**	**NG066331**	–
***Pseudocyclothyriellaclematidis***	**MFLU 16**-**0280**	**MT310596**	**MT214549**	–
***Pseudocyclothyriellaclematidis***	**MFLUCC 17**-**2177A**	**MT310595**	**MT214548**	**MT394730**
***Pseudodictyosporiumelegans***	**CBS 688.93**	**DQ018099**	**DQ018106**	–
*Pseudodictyosporiumindicum*	CBS 471.95	DQ018097	–	–
***Pseudodictyosporiumthailandica***	**MFLUCC 16**-**0029**	**KX259520**	**KX259522**	**KX259526**
***Pseudodictyosporiumwauense***	**NBRC 30078**	**DQ018098**	**DQ018105**	–
*Pseudodictyosporiumwauense*	DLUCC 0801	MF948622	MF948630	MF953165
***Verrucoccumcoppinsii***	**E00814291**	**MT918784**	**MT918770**	–
***Verrucoccumspribillei***	**SPO 2343**	**MT918780**	**MT918765**	–
*Vikalpaaustraliensis*	HKUCC 8797	DQ018092	–	–
***Vikalpagrandispora***	**KUNCC 22**-**12425**	**OP526638**	**OP526648**	**OP542240**
***Vikalpasphaerica***	**CGMCC 3.20682**	**OP526639**	**OP526649**	**OP542241**

## ﻿Results

### ﻿Phylogenetic analyses

The phylogram generated from maximum likelihood analysis based on combined ITS, LSU, and *tef*1-α sequence data represents the relationships of all genera of the Dictyosporiaceae. One hundred thirty-nine strains are included in the combined analyses, which comprise 2180 characters, including gaps (ITS: 1–470 bp, LSU: 471–1304 bp, *tef*1-α: 1305–2180 bp) after aligning, including *Periconiaigniaria* (CBS 379.86 and CBS 845.96) as the outgroup taxon. RAxML and Bayesian analyses were conducted and resulted in generally congruent topologies. The best RAxML tree with a final likelihood value of –21822.800488 is presented. The matrix had 996 distinct alignment patterns, with 25.73% undetermined characters or gaps. Estimated base frequencies were as follows: A = 0.233883, C = 0.255161, G = 0.273222, T = 0.237734; substitution rates AC = 1.700360, AG = 4.088659, AT = 2.394708, CG = 0.842455, CT = 9.208380, GT = 1.000000; gamma distribution shape parameter α = 0.226697. Statistical values for maximum likelihood (ML) above 75% and Bayesian posterior probabilities (PP) greater than 0.95 are given at the nodes.

Phylogenetic analyses showed that the new isolates were nested within Dictyosporiaceae and distributed in three genera, viz., *Dictyocheirospora*, *Dictyosporium*, and *Jalapriya*. *Dictyocheirosporanabanheensis* (KUNCC 23-15886) clustered with its type strain with 79% ML and 0.96 PP support. *Dictyocheirosporasplendida* formed a distinct lineage with *D.garethjonesii* and *D.nabanheensis* with 83% ML and 0.98 PP support. Two newly obtained strains of *Jalapriyaguttulata* (KUNCC 23-14576 and KUNCC 23-15861) clustered sister to *J.toruloides* with 99% ML and 1.00 PP support. *Dictyosporiumhughesii* (KUNCC 23-15923) and *Di.duliujiangense* (KUNCC 23-15949) clustered within the *Dictyosporium*. *Dictyosporiumduliujiangense* clustered sister to its type strain (GZCC 19-0426) with 100% ML and 1.00 PP support. The new isolate of *Di.hughesii* (KUNCC 23-15923) clustered with its type strain (KT 1847) with 97% ML and 1.00 PP support.

### ﻿Taxonomy

#### ﻿ Dictyocheirospora nabanheensis

Taxon classificationFungiPleosporalesDictyosporiaceae

Tibpromma & K.D. Hyde, Fungal Diversity 93: 10 (2018)

A9A98719-43A1-53DC-B961-40C212B41772

Index Fungorum: IF554474

Facesoffungi Number: FoF04483

Fig. 2

##### Description.

***Saprobic*** on submerged decaying wood in the Dulongjiang River. **Sexual morph** Undetermined. **Asexual morph**: Hyphomycetous. ***Colonies*** on natural substrate in small, scattered clusters, dark brown, velvety. ***Mycelium*** composed of immersed or partly superficial, hyaline to pale brown, septate, branched hyphae. ***Conidiophores*** micronematous, mononematous, septate, smooth, thin-walled, cylindrical, reduced to conidiogenous cells. ***Conidiogenous cells*** 8–13 × 3–5 μm (x̄ = 13.8 × 4.2 μm, n = 10), holoblastic, monoblastic, integrated, cylindrical, hyaline to pale brown, smooth-walled. ***Conidia*** 31–36 × 14–16 μm (x̄ = 33 × 15 μm, n = 36), solitary, ellipsoid to cylindrical, cheiroid, with a basal connecting cell, brown to yellow–brown, smooth-walled, euseptate, with discoid individual cells arranged in 6 compact rows (x̄ = 34.8 × 5.8 μm, n = 30) closely clustered at the apex, each row with 6–8 cells compactly and linearly clustered, with 1–2 rounded to cylindrical hyaline, 6–10 × 5–7 μm, appendages arising from near the middle of conidial rows.

##### Culture characteristics.

Conidia germinating on WA within 12 h and germ tubes were produced at the basal region (Fig. 2f). Colonies on PDA at 22 °C, circular, white at margin around the edge and light gray in the center, raised on surface, with white, pale orange concentric rings, yellow–orange in the middle on the reverse side.

##### Material examined.

China • Yunnan Province, Dulongjiang River, on submerged decaying wood, 2 May 2023 (Altitude: 1422 m, 27.793592°N, 98.330416°E), Ying Wang, S4719 (KUN-HKAS 135951), living culture, KUNCC 23-15886.

##### Notes.

**Figure 1.**
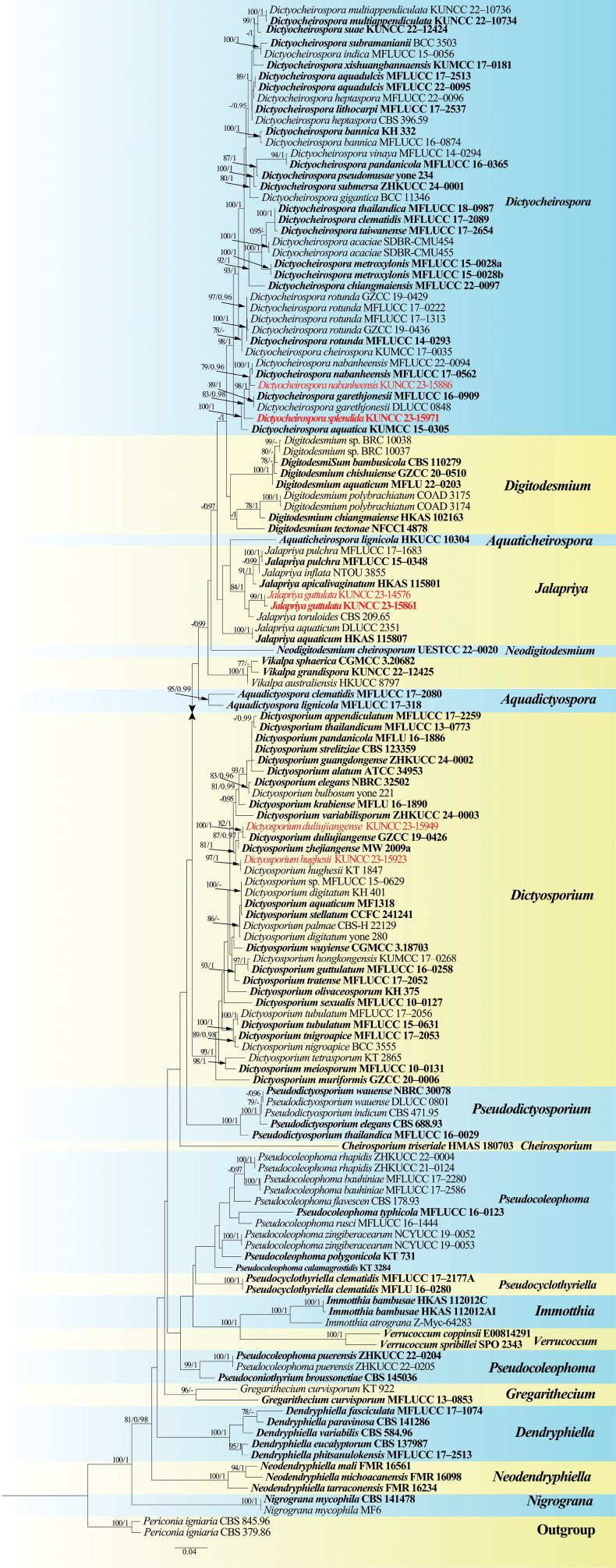
The maximum likelihood (ML) tree is based on the combined ITS, LSU, and *tef*1-α sequence data. Bootstrap support values with ML greater than 75% and Bayesian posterior probabilities (PP) greater than 0.95 are given above the nodes, shown as “ML/PP.” The tree was rooted to *Periconiaigniaria* (CBS 379.86 and CBS 845.96). New species are indicated in red, and type strains are in bold.

The newly collected fungus has closely clustered terminal cells at the apex of the conidia, which is similar to those of the genus *Dictyocheirospora*. Phylogenetic analysis of the combined ITS, LSU, and *tef*1-α sequence data showed that the new strain KUNCC 23-15886 clustered sister to the ex-type strain of *D.nabanheensis* (KUMCC 16-0152) with 79% ML and 0.96 PP statistical support (Fig. 1). Morphologically, KUNCC 23-15886 resembles *D.nabanheensis* in having conidia with hyaline, globose to subglobose appendages in the middle region. *Dictyocheirosporanabanheensis* (KUMCC 16-0152) differs from KUNCC 23-15886 in having slightly larger conidia (38 × 20 μm vs. 33 × 15 μm) (Tibpromma et al. 2018a; Shen et al. 2022b) and with more cells in each row (6–10 cells vs. 6–8 cells) (Tibpromma et al. 2018a; Shen et al. 2022b). However, there are only 0.83% (4/480) differences between ITS sequence data. Therefore, we identified our collection as *D.nabanheensis*. The type of *D.nabanheensis* was found on dead leaves of *Pandanus* sp. in a terrestrial habitat in Yunnan, China, and the strain of MFLUCC 22-0094 was found on submerged decaying wood in a freshwater habitat in Thailand. Our study isolated this species for the first time on submerged decaying wood in Yunnan province, China.

**Figure 2.**
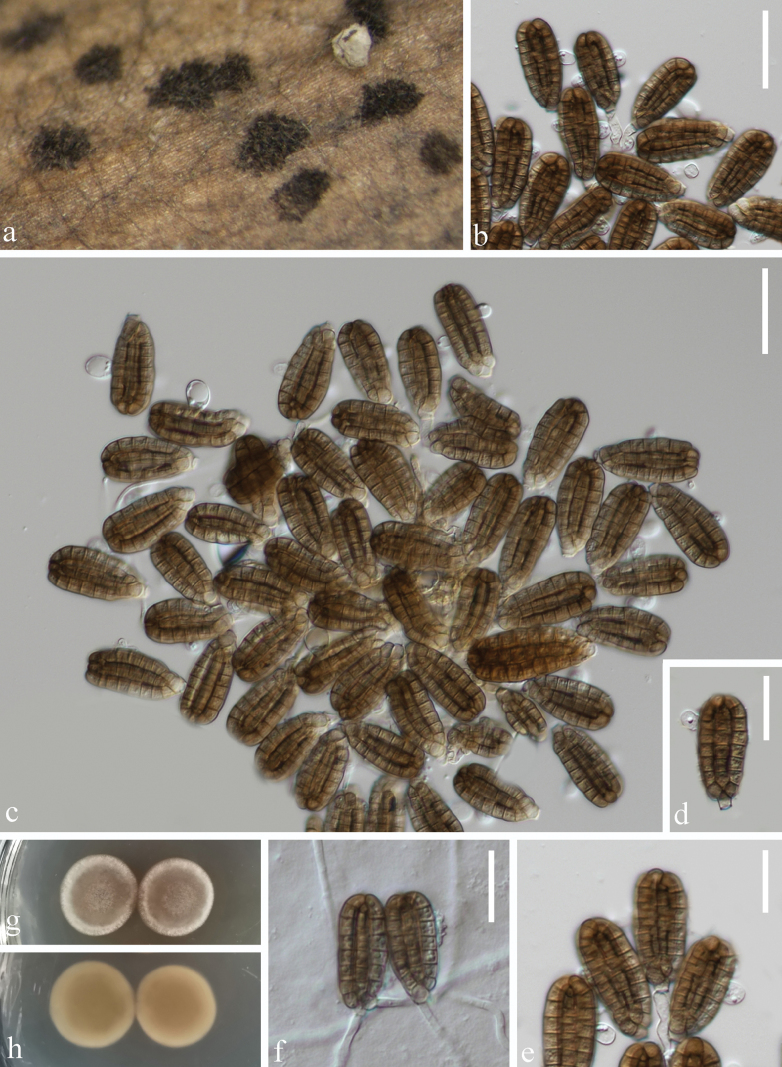
*Dictyocheirosporanabanheensis* (KUN-HKAS 135951) **a** colonies on submerged decaying wood **b, c** conidiophores and conidia **d–e** conidia **f** germinated conidia; Culture on PDA from front (**g**) and reverse (**h**). Scale bars: 30 µm (**b–c**); 20 µm (**d, e, f**).

#### ﻿ Dictyocheirospora splendida

Taxon classificationFungiPleosporalesDictyosporiaceae

Y. Wang & Z.L. Luo sp. nov.

C8CD1EDC-4317-5F20-8388-DD7E679654B1

Fungal Names: FN 572104

Fig. 3

##### Etymology.

Referring to the glistening colony on natural substrates.

##### Holotype.

KUN-HKAS 135954.

##### Description.

***Saprobic*** on submerged decaying wood. **Sexual morph**: Undetermined. **Asexual morph**: Hyphomycetous. ***Colonies*** glistening on natural substrate, in small pulvinate groups, scattered on surface, dark brown, velvety. ***Mycelium*** immersed on substrate, composed of brown, smooth, septate, branched hyphae. ***Conidiophores*** micronematous, mononematous, hyaline to pale brown, 4–5 μm wide, mostly reduced to conidiogenous cells. ***Conidiogenous cells*** 6–11 × 3–6 μm (x̄ = 8.5 × 4.7 μm, n = 10), holoblastic, integrated, terminal, cylindrical, hyaline to pale brown, smooth-walled. ***Conidia*** 37–43 × 14–17 μm (x̄ = 40 × 16 μm, n = 30) solitary, acrogenous, cheiroid, with a basal connecting cell, brown to yellow-brown, arranged in 6 compact rows closely clustered at the apex, with each row composed of 6–9 cells, euseptate, with one appendage at the center of conidia.

##### Culture characteristics.

**Figure 3.**
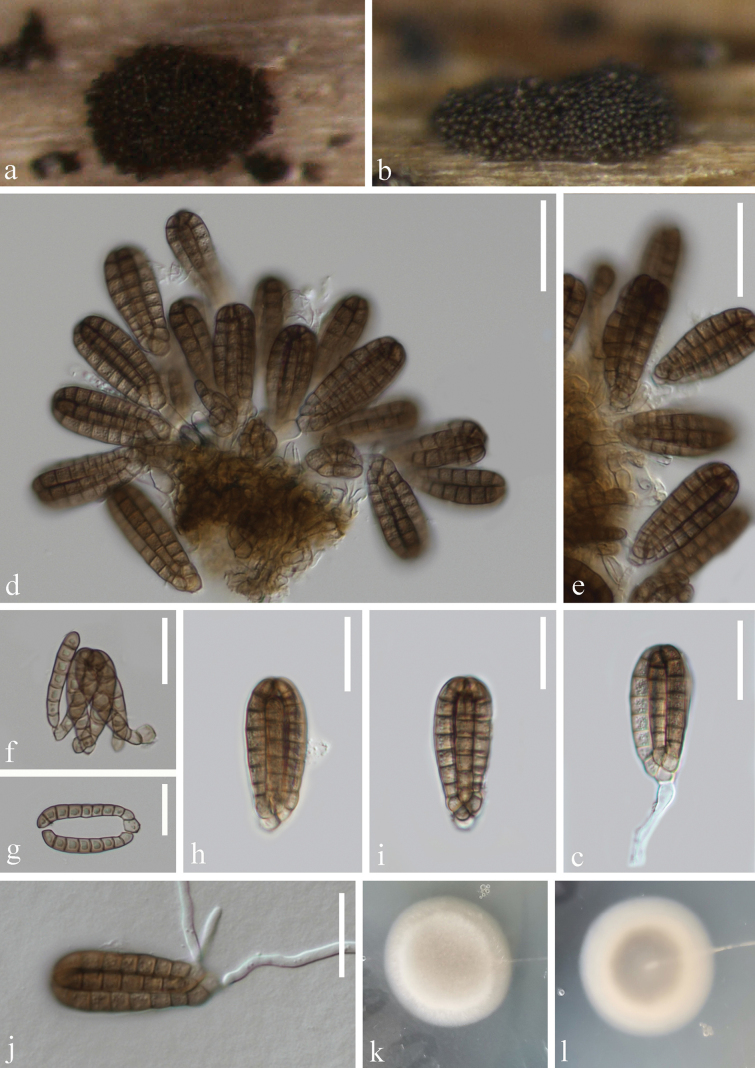
*Dictyocheirosporasplendida* (KUN-HKAS 135954, holotype) **a, b** colonies on submerged decaying wood **c–e** conidiophores with conidia **f** crushed single conidia **g** rows of conidial cells **h, i** conidia **j** germinated conidium; Culture on PDA from front (**k**) and reverse (**l**). Scale bars: 30 µm (**d, e**); 20 µm (**c, f–j**).

Conidia germinating on PDA within 12 h and germ tubes produced at the base (Fig. 3k). Colonies grew on PDA at 22 °C, circular, white at margin and light gray in the center, slightly raised on surface cottony, with mycelium partially infiltrating into the culture and white on the edge, grayish-white in the middle on the reverse side.

##### Material examined.

China • Yunnan Province, Dulongjiang River, on submerged decaying wood, 2 May 2023 (Altitude: 1418 m, 27.783341°N, 98.330741°E), Ying Wang, S4957 (KUN-HKAS 135954, holotype); ex-type culture, KUNCC 23-15971.

##### Notes.

In the phylogenetic analyses, *Dictyocheirosporasplendida* was basal to *D.garethjonesii* and *D.nabanheensis*, with 83% ML and 0.98 PP support (Fig. 1). The new strain KUNCC 23-15971 has 2.29% (11/480 bp) ITS and 3.14% (26/829 bp) *tef*1-α base pair difference from *D.nabanheensis* (MFLUCC 17-0562) as well as 2.55% (13/509 bp) ITS and 2.55% (31/874 bp) *tef*1-α base pair difference from *D.garethjonesii* (DLUCC 0848). However, LSU sequences of these three species are identical. Morphologically, *D.splendida* has sporodochial conidiomata, darkly pigmented, micronematous or semi-macronematous conidiophores, and acrogenous, cheiroid, and brown-colored conidia, which match the characteristics of *Dictyocheirospora* (Boonmee et al. 2016). However, *D.nabanheensis* exhibits distinct conidial appendages (Tibpromma et al. 2018b), which were inconspicuous in *D.splendida*. In addition, *D.splendida* exhibits a significant difference in conidial size compared to *D.garethjonesii* (40 × 16 μm vs. 50 × 20 μm) (Boonmee et al. 2016). Based on morphological and phylogenetic analysis, we introduce *Dictyocheirosporasplendida* as a new species.

#### ﻿ Dictyosporium duliujiangense

Taxon classificationFungiPleosporalesDictyosporiaceae

L.L. Liu & Z.Y. Liu, Phytotaxa 606: 266 (2023)

97BD841D-918E-54E5-8BCC-6C065AE1D0CA

Index Fungorum: IF900403

Facesoffungi Number: FoF14119

Fig. 4

##### Description.

***Saprobic*** on submerged decaying wood. **Sexual morph**: Undetermined. **Asexual morph**: Hyphomycetous. ***Colonies*** on natural substrate punctiform, sporodochial, scattered, dark brown to black, velvety, glistening. ***Mycelium*** mostly immersed, composed of septate, branched, hyaline to pale brown hyphae. ***Conidiophores*** macronematous, branched, septate, cylindrical, hyaline to pale yellow, rough-walled, 3–5 μm wide, sometimes reduced to conidiogenous cells. ***Conidiogenous cells*** 11–18 × 3–5 μm (x̄ = 15 × 4 μm, n = 17) monoblastic, integrated, terminal, determinate, pale yellow to yellow. ***Conidia*** 38–45 × 22–26 μm (x̄ = 41.5 × 24 μm, n = 30), acrogenous, solitary, cheiroid, smooth-walled, complanate, yellowish-brown to pale brown, range from moderate orange at the base to pale yellow at the apex when immature, consisting of five closely adpressed rows of cells 33–38 ×4–6 μm (x̄ = 35.5 × 5.5 μm, n = 30), side rows shorter than middle rows, 4–10-euseptate in each row of cells, constricted at septa, with one or two hyaline, tubular, elongated appendages, narrower in the middle, 42–59 × 6–9 μm (x̄ = 50.2 × 7.2 μm, n = 28) containing small guttules, suspended from the apical part of the outer rows of cells.

##### Cultural characteristics.

Conidia germinating on PDA within 24 h and germ tubes produced from the apex. Colonies grew on PDA at 22 °C, circular, with fluffy, dense, white mycelium. The reverse side has a pale-yellow edge and an orange center.

**Figure 4.**
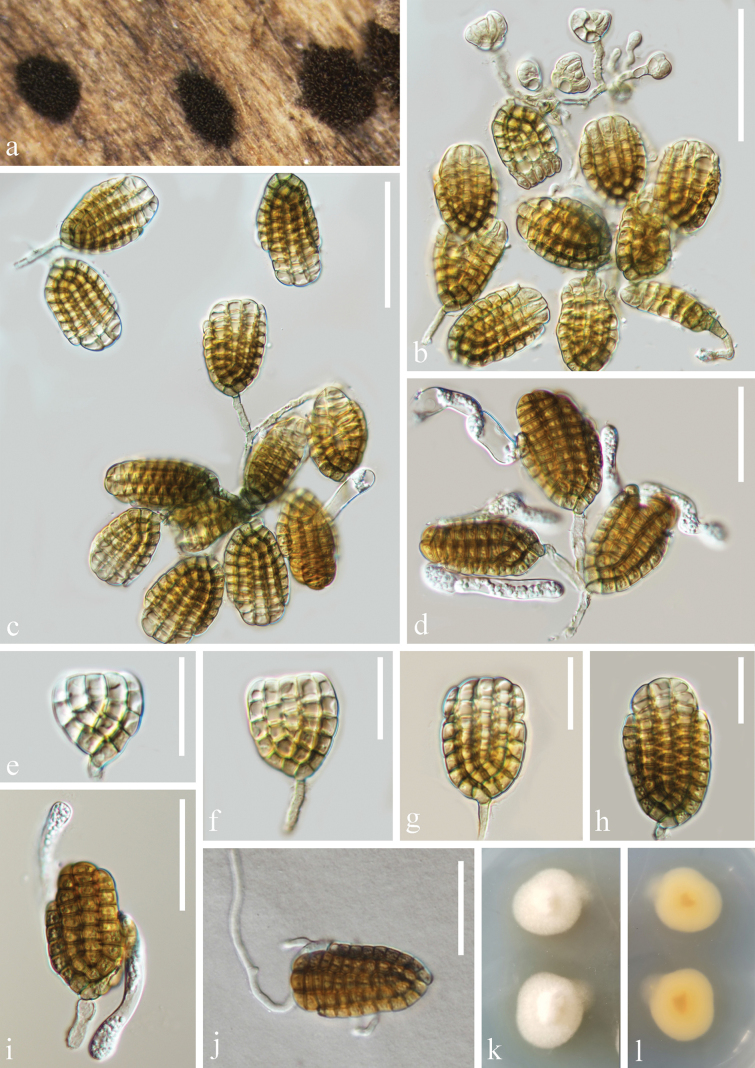
*Dictyosporiumduliujiangense* (KUN-HKAS 135956) **a** colonies on submerged decaying wood **b–d** conidiophores with conidia **e–i** conidia and conidiogenous cells **j** germinated conidium; Culture on PDA from front (**k**) and reverse (**l**). Scale bars: 50 µm (**b–c**); 30 µm (**d, i–j**); 20 µm (**e–h**).

##### Material examined.

China • Yunnan Province, Dulongjiang River, on submerged decaying wood, 2 May 2023 (Altitude: 1516 m, 27.686450°N, 98.349933°E), Ying Wang, S4923 (KUN-HKAS 135956), living culture, KUNCC 23-15949.

##### Notes.

The conidia in *Dictyosporium* are complanate and not closely clustered at the apex (Boonmee et al. 2016; Yang et al. 2018). The morphological characteristics of the newly collected strain (KUNCC 23-15949) match with *Dictyosporium* species (Boonmee et al. 2016, Yang et al. 2018). Based on the phylogenetic analyses of the combined ITS, LSU, and *tef*1-α sequence data, our new strain (KUNCC 23-15949) shows a close affinity to *Di.duliujiangense* (GZCC 19-0426). There is a 1.56% (8/512) difference in ITS sequence data between KUNCC 23-15949 and GZCC 19-0426, but the LSU and *tef*1-α sequences of these two species are identical. Morphologically, our collection differs from the type material in the size of conidia (41.5 × 24 μm vs. 33 × 20 μm) and the length of the appendages (50.2 × 7.2 μm vs. 27 × 6 μm) (Liu et al. 2023). *Di.duliujiangense* was first reported on a decaying branch submerged in a freshwater river in Dushan County, Guizhou Province, China (Liu et al. 2023). Based on these findings, we identified our collection as *Di.duliujiangense*.

#### ﻿ Dictyosporium hughesii

Taxon classificationFungiPleosporalesDictyosporiaceae

McKenzie, Mycotaxon 111: 156 (2010)

68FE10B1-503B-5530-8BFB-1767902AE9B3

515243

Fig. 5

##### Description.

***Saprobic*** on submerged decaying wood. **Sexual morph**: Undetermined. **Asexual morph**: Hyphomycetous. ***Colonies*** on natural substrate, punctiform, sporodochia, scattered but coalescing, black, irregular. ***Mycelium*** immersed, pale to brown. ***Conidiophores*** micronematous, mononematous, subhyaline, thin-walled, smooth. ***Conidiogenous cells*** holoblastic, determinate, cylindrical, 3.5–5 µm wide. ***Conidia*** 56–68 × 23–29 μm (x̄ = 62 × 26.3 μm, n = 40), solitary, acrogenous, medium brown, complanate, ellipsoidal or cylindrical, cheiroid, arranged in 7 closely adpressed rows of cells, with outer rows of cells arising from a basal cell, the side rows lower than middle rows, outer rows extending about two-thirds of the way along the conidium, each row containing 6–12 cells, constricted at septa, slightly thickened walls and septa, with or without appendages.

##### Cultural characteristics.

Conidia germinating on PDA within 24 h and germ tubes produced from the basal cells and the apex of the row of cells. Colonies grew on PDA at 22 °C, circular, with fluffy mycelium; the mycelium is dense in the center and sparse at the edges, white at the outer margin and light yellow at the center, light yellow on the reverse side.

**Figure 5.**
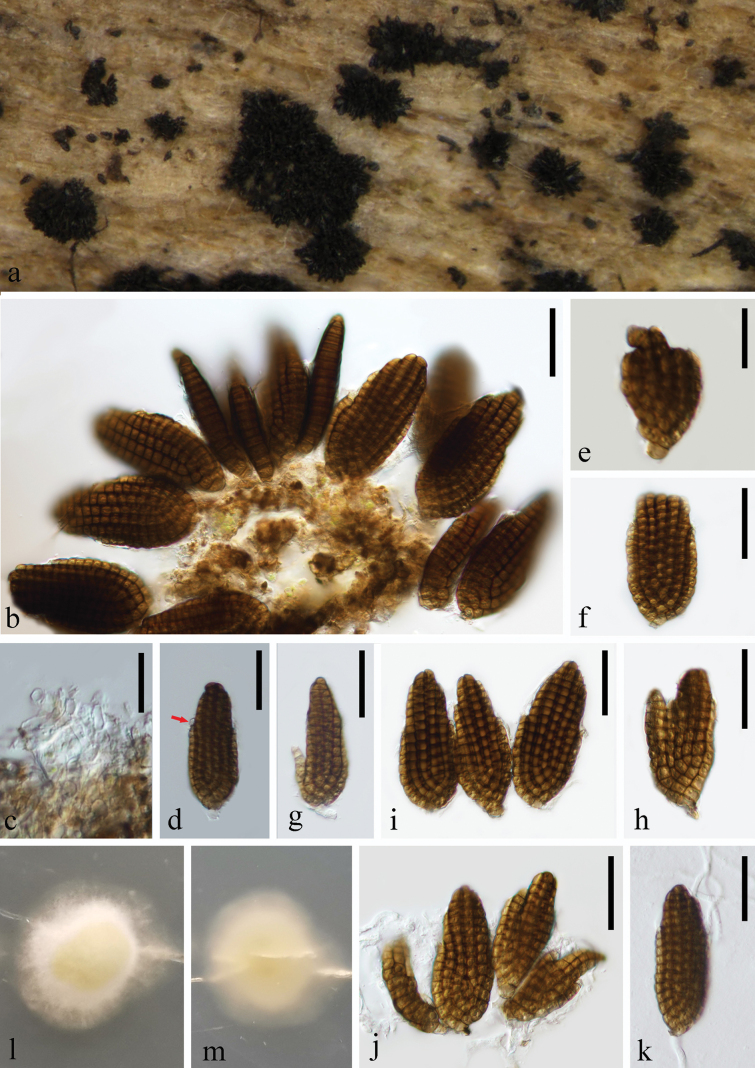
*Dictyosporiumhughesii* (KUN-HKAS 135963) **a** colonies on submerged decaying wood **b** conidia **c** conidiophores and Conidiogenous cells **d** conidia with appendages **e–j** conidia **k** germinated conidium; Culture on PDA from front (**l**) and reverse (**m**). Scale bars: 30 µm (**b, d, f–k**); 20 µm (**c, e**).

##### Material examined.

China • Yunnan Province, Dulongjiang River, on submerged decaying wood, 2 May 2023 (Altitude: 1418 m, 27.783341°N, 98.330741°E), Ying Wang, S4812 (KUN-HKAS 135963), living culture, KUNCC 23-15923.

##### Notes.

This collection was identified as *Dictyosporiumhughesii* based on the phylogenetic analysis and morphological characteristics. Phylogenetic analysis of the combined ITS, LSU, and *tef*1-α sequence data showed that our new strain (KUNCC 23-15923) clustered with the ex-type strain of *Di.hughesii* (KT 1847) with 97% ML and 1.00 PP support (Fig. 1). Morphologically, the conidia of our new collection are slightly larger (56–68 µm vs. 40–47 µm), and the appendages are not prominent. *Di.hughesii* has been found on dead leaves of *Rhopalostylissapida* in New Zealand (McKenzie 2010) and on dead branches of *Stewartiamonadelpha* in Japan (Ghosh et al. 2015; Tanaka et al. 2015), and herein it is reported from submerged decaying wood in a freshwater habitat in China.

#### ﻿ Jalapriya guttulata

Taxon classificationFungiPleosporalesDictyosporiaceae

Y. Wang & Z.L. Luo sp. nov.

981A4DC5-937A-53AF-8745-6334EE6B9703

Fungal Names: FN 572106

Fig. 6

##### Etymology.

Referring to the guttulate cells of the conidia.

##### Holotype.

KUN-HKAS 135959.

##### Description.

**Figure 6.**
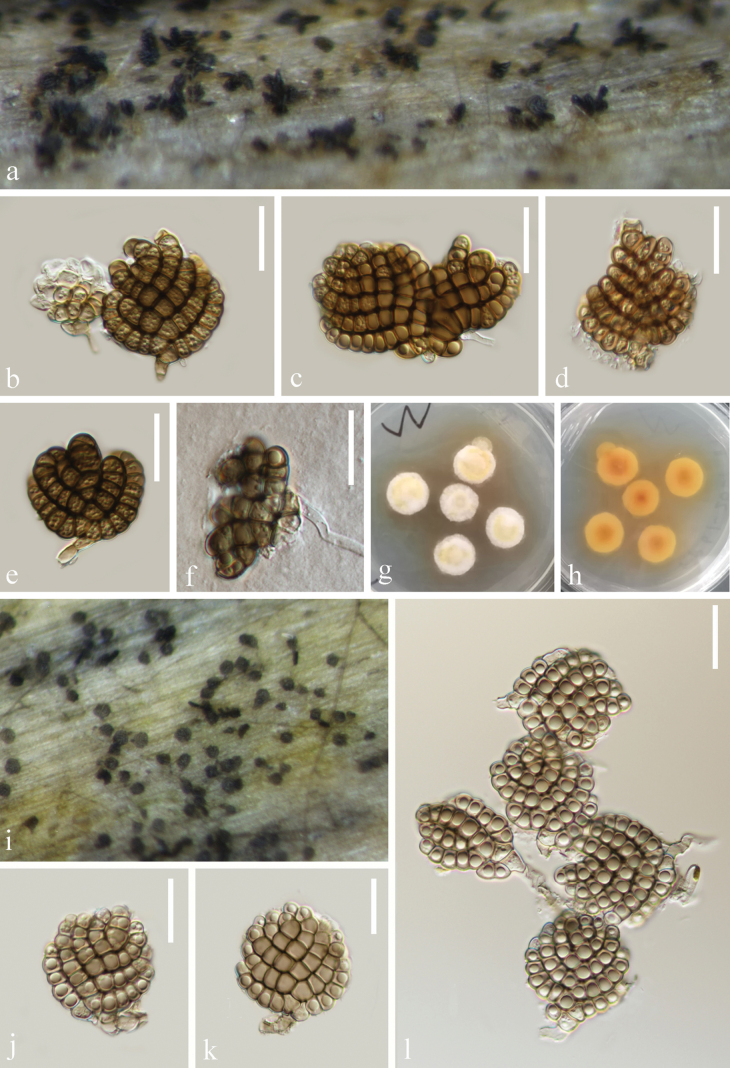
*Jalapriyaguttulata* (**a–h**KUN-HKAS 135959, holotype; **i–l**KUN-HKAS 135948) **a**, **i** colonies on submerged decaying wood **b–e, j–l** conidiophores and conidia **f** germinated conidia; Culture on PDA from front (**g**) and reverse (**h**). Scale bars: 20 µm (**b–f, j–l**).

***Saprobic*** on submerged decaying wood. **Sexual morph**: Undetermined. **Asexual morph**: Hyphomycetous. ***Colonies*** on natural substrate effuse, scattered, dark brown to black. ***Mycelium*** mostly immersed, partly superficial, composed of smooth, septate, branched, pale brown to brown, or pale orange. ***Conidiophores*** micronematous, unbranched, thin-walled, smooth, 3–4 μm wide. ***Conidiogenous cells*** holoblastic, integrated, terminal, pale brown to brown, or pale brown, thin-walled. ***Conidia*** 31–43 × 25–38 μm (x̄ = 37.3 × 31.5 μm, n = 30) (KUNCC 23-15861) or 34–41 × 30–37 μm (x̄ = 37.5 × 33.7 μm, n = 30) (KUNCC 23–14576), acrogenous, septate, distinctly constricted at septa, holoblastic, solitary or paired (Fig. 6c), palmate, light olive-brown when immature and olive-brown to reddish-brown or pale brown, with some cells slightly darker in the center and lighter at the edges when mature. with 5–11 overlapping rows of unequal length and cells arranged in a plane, 1–2 outer rows arising from a basal cell, with additional rows arising from the base of the previous row, these rows are slightly curved inward, with each row consisting of 2–9 cells, with number of cells gradually decreasing from base to upward except for basal row of cells, with some cells in the uppermost row enlarged.

##### Culture characteristics.

Conidia germinating on PDA within 24 h and germ tubes produced at the basal cell and the apex of the conidium. Colonies grew on PDA at 22 °C, circular, mycelium was loose, flocculent, on the obverse, at first white, later becoming light yellow. The reverse side is orange, with the center being the darkest.

##### Material examined.

China • Yunnan Province, Dulongjiang River, on submerged decaying wood, 2 May 2023, Fig. 6a–f: (Altitude: 1397 m, 27.702847°N, 98.349787°E), Ying Wang, S4558 (KUN-HKAS 135959, holotype); ex-type culture, KUNCC 23-15861. Fig. 6i–l: (Altitude: 1702 m, 28.033444°N, 98.317358°E), Ying Wang, S4587 (KUN-HKAS 135948), living culture, KUNCC 23–14576.

##### Notes.

Six species are currently accepted in *Jalapriya*, among which *J.inflata* and *J.toruloides* have been reported from wood in marine habitats (Goh et al. 1999; Kirschner et al. 2013; Tibell et al. 2020; Du et al. 2025). These two species were originally classified under *Dictyosporium* and were transferred to the genus *Jalapriya* by Boonmee et al. (Boonmee et al. 2016). *J.apicalivaginatum*, *J.aquaticum*, and *J.pulchra* were found on submerged decaying wood in freshwater habitats, the first one from Henan province and the latter two from Yunnan province (Boonmee et al. 2016; Fu et al. 2021). While *J.cheirospora* was found on dead stems of the medicinal plant *Disporumlongistylum* in Kunming City, Yunnan Province, China. In this study, we collected two morphologically similar strains (KUNCC 23-15861 and KUNCC 23–14576) from submerged decaying wood. Despite the differences in conidial color (olive-brown to reddish-brown vs. pale brown to moderate brown) and size (37.3 × 31.5 μm vs. 37.5 × 33.7 μm), both strains share similar characteristics in culture and natural colonies, have guttulate conidia, and relatively few cells in the terminal cell row. Notably, no differences were found in the sequences of ITS and *tef*1-α gene regions between KUNCC 23-15861 and KUNCC 23–14576. Which indicated that the two strains belong to the same species.

Based on the phylogenetic analyses of the combined ITS, LSU, and *tef*1-α sequence data, our new strains (KUNCC 23-15861 and KUNCC 23-14576) clustered with *J.toruloides* (CBS 209.65) with 99% ML and 1.00 PP support (Fig. 1). The ITS sequence of *J.guttulata* has a 2.7% (14/518 bp) difference from *J.toruloides* (CBS 209.65). But the LSU sequence has only one base pair (1/494 bp) difference from *J.toruloides*. Morphologically, *J.guttulata* has more conidial rows of cells (5–11 rows vs. 6–8 rows); the conidia of *J.guttulata* are shorter (37.3–37.5 μm vs. 38–56 μm) and wider (31.5–33.7 μm vs. 25–24 μm) than *J.toruloides*; the rows of these two species are of unequal length, and the apical rows of the conidia in most *J.guttulata* have fewer cells (Henningsson 1974; Goh et al. 1999). Notably, solitary or paired conidia of *J.guttulata* were observed for the first time in the genus *Jalapriya*. Based on morphological characteristics and phylogenetic analysis, we identify our isolates as a new species.

## ﻿Discussion

Dulongjiang River is rich in lignicolous freshwater fungi. To date, 18 lignicolous freshwater fungi have been described and illustrated from this river, including 11 new species (Su et al. 2016; Wang et al. 2016; Luo et al. 2017, 2018b, 2019; Bao et al. 2018; Li et al. 2020). It is worth continuing the investigation on the diversity of microfungi in the Dulongjiang river basin. Dulongjiang River is particularly rich in species of Dictyosporiaceae. Wang et al. (2016) introduced a new species, *Dictyocheirosporaaquatica*, from the Dulongjiang river basin. In the present study, we reported five Dictyosporiaceae species.

The conidia of *Jalapriya* are typically solitary (Boonmee et al. 2016; Tibell et al. 2020; Fu et al. 2021), whereas in this study paired conidia were observed for the first time. Notably, the overall morphologies of KUNCC 23-15861 and KUNCC 23-14576 differ significantly. However, their sequence data showed no differences which indicates that it is difficult to accurately identify species based solely on morphology, and combining both morphological and phylogenetic analyses is essential when identifying species.

Since the early 2000s, mycologists have been working on the diversity of Dictyosporiaceae. However, early studies relied heavily on the morphological characters; only a few species were associated with molecular data, resulting in the taxonomic status of numerous species remaining inaccurate (Luo et al. 2004). Over the past decade, the family has been studied intensively based on both morphology and multi-gene (ITS, LSU, and *tef*1-α) phylogenetic analyses; as a result, the taxonomy of several species has been revised (Boonmee et al. 2016; Yang et al. 2018). ITS and *tef*1-α sequences play a crucial role in the identification of species within the Dictyosporiaceae. The comparison revealed significant nucleotide differences between the ITS and *tef*1-α sequences among different species. However, many species lack protein-coding genes (*tef*1-α) sequences. As the number of species increases, it becomes more difficult to distinguish different species based on ITS and LSU sequences alone. For instance, *Dictyosporiumaquaticum* and *Di.stellatum* only have ribosomal sequences (ITS and LSU) available in GenBank; although there are significant morphological differences, they cannot be distinguished on the phylogenetic tree (Crous et al. 2011; Liu et al. 2015; Shen et al. 2022b; Shu et al. 2024). Therefore, it is necessary to add protein-coding genes for identifying species in future studies.

Dictyosporiaceae has been accommodated as a pleomorphic group and is recently being well-studied based on the combination of morphological and phylogenetic analysis. However, there are still some unsettled issues within the family (Tennakoon et al. 2023; Shu et al. 2024). The phylogenetic distinctions between *Immotthia*, *Pseudocoleophoma*, *Pseudoconiothyrium*, *Pseudocyclothyriella*, and *Verrucoccum* are still ambiguous (Shen et al. 2022b; Liu et al. 2023). Whereas *Pseudocoleophoma* is polyphyletic, forming three clades within the family (Feng et al. 2024). Therefore, integrated further research on this family is urgently necessary.

## Supplementary Material

XML Treatment for Dictyocheirospora nabanheensis

XML Treatment for Dictyocheirospora splendida

XML Treatment for Dictyosporium duliujiangense

XML Treatment for Dictyosporium hughesii

XML Treatment for Jalapriya guttulata

## ﻿Additional information

### Author ORCIDs

Ying Wang https://orcid.org/0009-0004-1995-1671

D. Jayarama Bhat https://orcid.org/0000-0002-3800-5910

Dan-Feng Bao https://orcid.org/0000-0002-5697-4280

Hong-Wei Shen https://orcid.org/0000-0003-2508-1970

Zong-Long Luo https://orcid.org/0000-0001-7307-4885

### Data availability

All of the data that support the findings of this study are available in the main text.
